# Supplementary material for: Mechanochemical Synthesis of PdO2 Nanoparticles Immobilized over Silica Gel for Catalytic Suzuki–Miyaura Cross-Coupling Reactions Leading to the C-3 Modification of 1H-Indazole with Phenylboronic Acids
Source: Molecules. 2023 Oct 20;28(20):7190. doi: 10.3390/molecules28207190 (PMC10609228; doi:10.3390/molecules28207190)
Supplement: Supplementary file 1 [file molecules-28-07190-s001.zip › molecules-2623883-Supplementary Materials.pdf]

Supplementary Materials

Mechanochemical Synthesis of PdO_2 Nanoparticles Immobilized Over Silica Gel for Catalytic Suzuki–Miyaura Cross-Coupling Reactions Leading to the C-3 Modification of 1*H*-Indazole with Phenylboronic Acids

Qin Pan [1,2,3], Yong Wu [1,2,3], Aqun Zheng [1], Xiangdong Wang [1,2,3], Xiaoyong Li [1,2,3], Wanqin Wang [1,2,3], Min Gao [1,2,3], Zainab Bibi [1], Sidra Chaudhary [1], Yang Sun [1,2,3,*]

[1] Department of Applied Chemistry, School of Chemistry, Xi'an Jiaotong University, No. 28, Xianning West Road, Xi'an 710049, P.R. China

[2] Xi'an Biomass Green Catalysis and Advanced Valorization International Science and Technology Cooperation Base, No. 28, Xianning West Road, Xi'an 710049, P.R. China

[3] Xixian New District Xingyi Advanced Materials Technology Co., Ltd., Room 1046, 1st floor, Hongdelou, Building No. 20, Science and Technology Innovation Port, Western China, Fengxi New City, Xixian New District, Xi'an 712000, Shaanxi Province, P.R. China

* Corresponding author. Tel.: +86 29 82663914 (Y. Sun); fax: +86 29 82668559 (Y. Sun).
E-mail address: sunyang79@mail.xjtu.edu.cn (Y. Sun).

Table of Contents

S1. XPS measurement of Si 2p region for synthesized catalyst (Figure S1)
S2. XPS measurement of P 2p region for C3 (Figure S2)
S3. FT-IR spectra of synthesized catalysts (Figure S3)
S4. UV-Vis spectra of synthesized catalysts (Figure S4)
S5. ^{1}H NMR spectrum of 3-iodo-1*H*-indazole (Figure S5)
S6. ^{13}C NMR spectrum of 3-iodo-1*H*-indazole (Figure S6)
S7. ^{1}H NMR spectrum of *tert*-butyl-3-iodo-1*H*-indazole-1-carboxylate (Figure S7, full

scale)

S8. ^{1}H NMR spectrum of *tert*-butyl-3-iodo-1*H*-indazole-1-carboxylate (Figure S8, aromatic region)

S9. ^{1}H NMR spectrum of *tert*-butyl-3-(3-(methoxycarbonyl)phenyl)-1*H*-indazole-1-carboxylate (Figure S9, full scale)

S10. ^{1}H NMR spectrum of *tert*-butyl-3-(3-(methoxycarbonyl)phenyl)-1*H*-indazole-1-carboxylate (Figure S10, aromatic region)

S11. ^{1}H NMR spectrum of *tert*-butyl-3-(4-(methoxycarbonyl)phenyl)-1*H*-indazole-1-carboxylate (Figure S11, full scale)

S12. ^{1}H NMR spectrum of *tert*-butyl-3-(4-(methoxycarbonyl)phenyl)-1*H*-indazole-1-carboxylate (Figure S12, aromatic region)

S1. XPS measurement of Si 2p region for synthesized catalyst

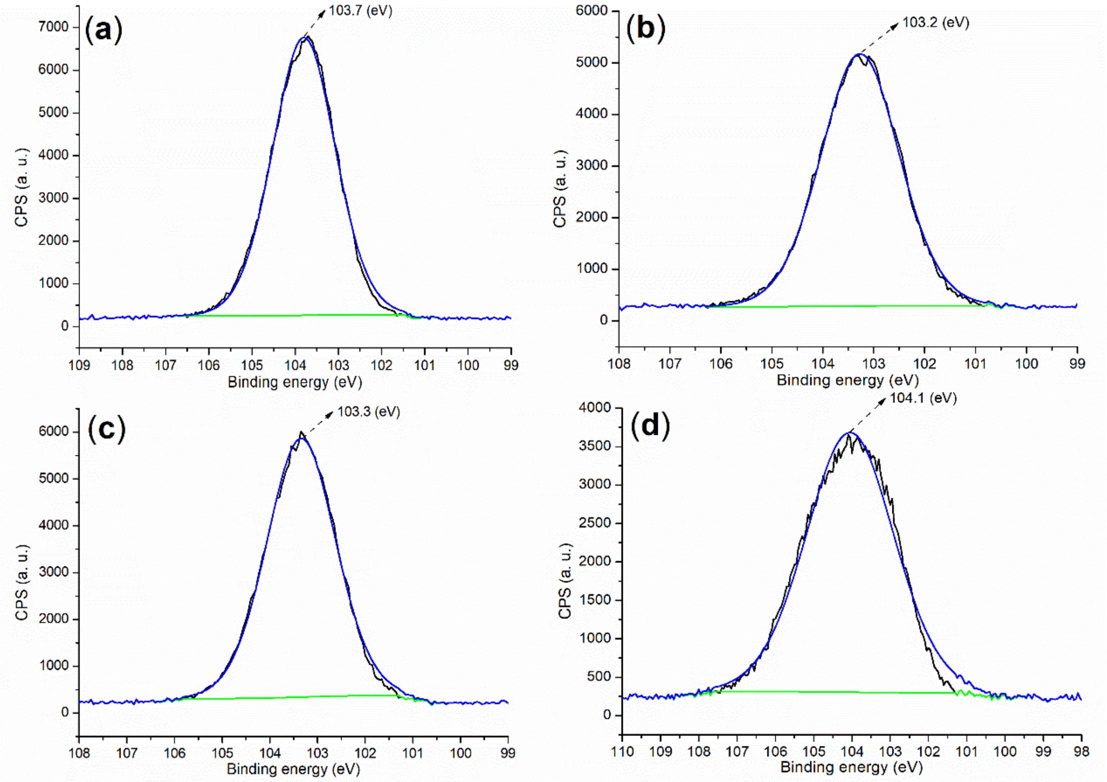

Figure S1. XPS measurement of Si 2p region for synthesized catalyst: (**a**) C0, (**b**) C1, (**c**) C2, (**d**) C3.

S2. XPS measurement of P 2p region for C3

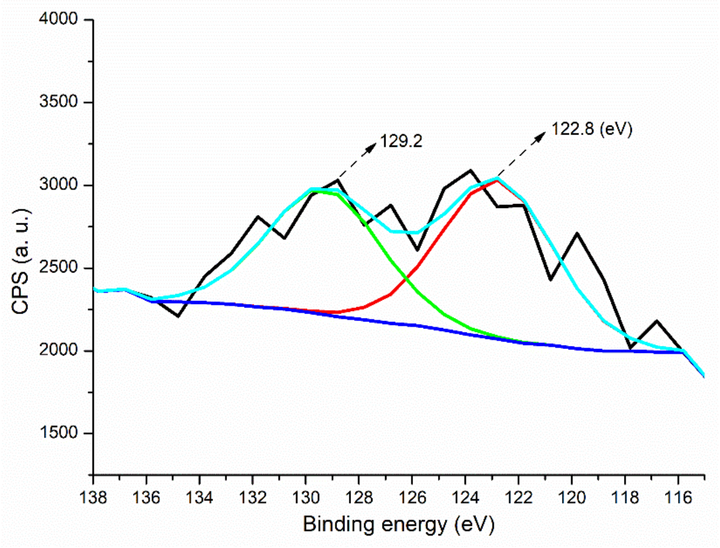

Figure S2. XPS measurement of P 2p region for C3.

S3. FT-IR spectra of synthesized catalysts

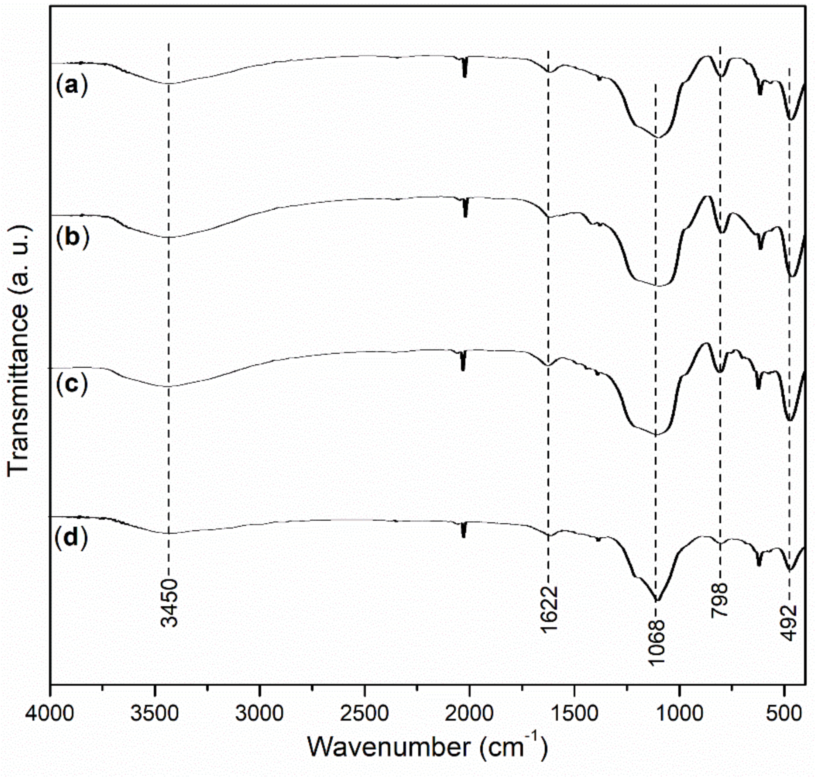

Figure S3. FT-IR spectra of synthesized catalysts: (**a**) C0, (**b**) C1, (**c**) C2, (**d**) C3.

S4. UV-Vis spectra of synthesized catalysts

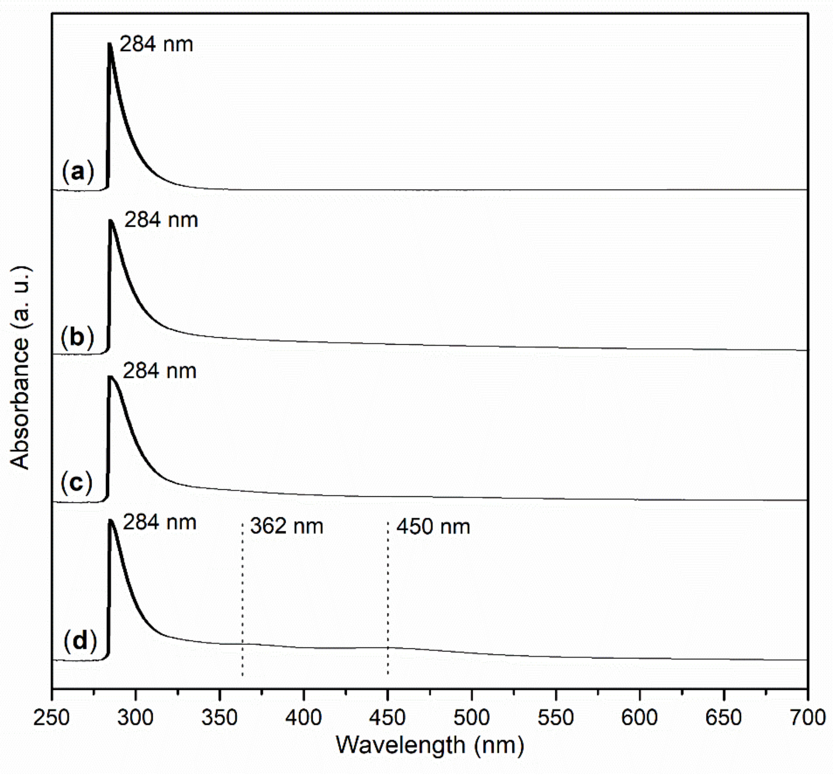

Figure S4. UV-Vis spectra of synthesized catalysts: (**a**) C0, (**b**) C1, (**c**) C2, (**d**) C3.

S5. ^{1}H NMR spectrum of 3-iodo-1*H*-indazole

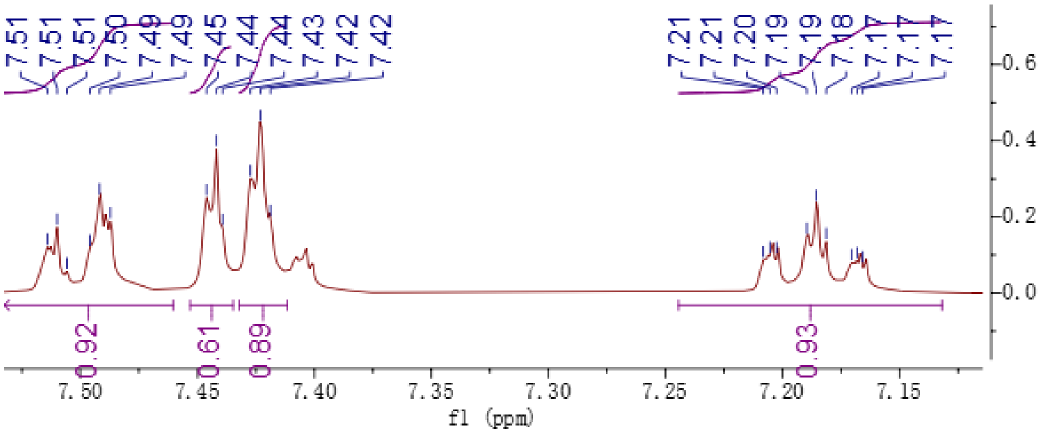

Figure S5. ^{1}H NMR spectrum of 3-iodo-1*H*-indazole.

S6. ^{13}C NMR spectrum of 3-iodo-1*H*-indazole

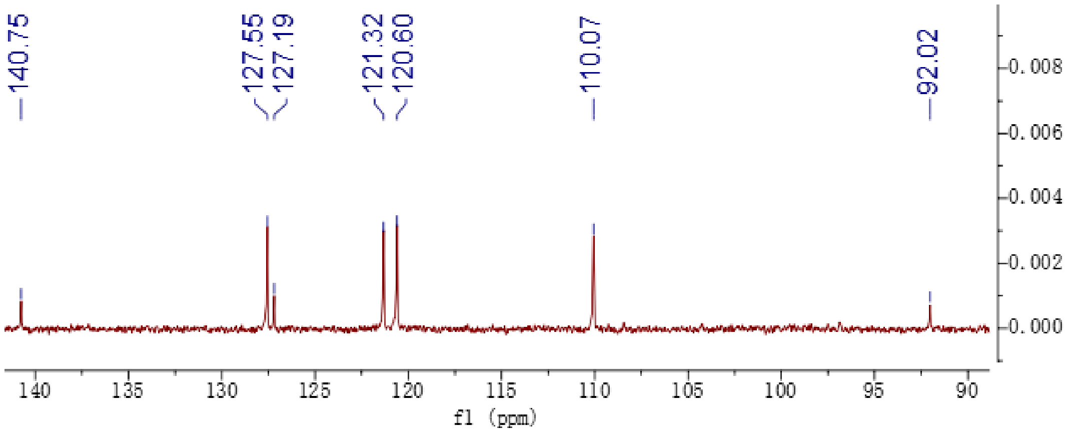

Figure S6. ^{13}C NMR spectrum of 3-iodo-1*H*-indazole.

S7. ^{1}H NMR spectrum of *tert*-butyl-3-iodo-1*H*-indazole-1-carboxylate (full scale)

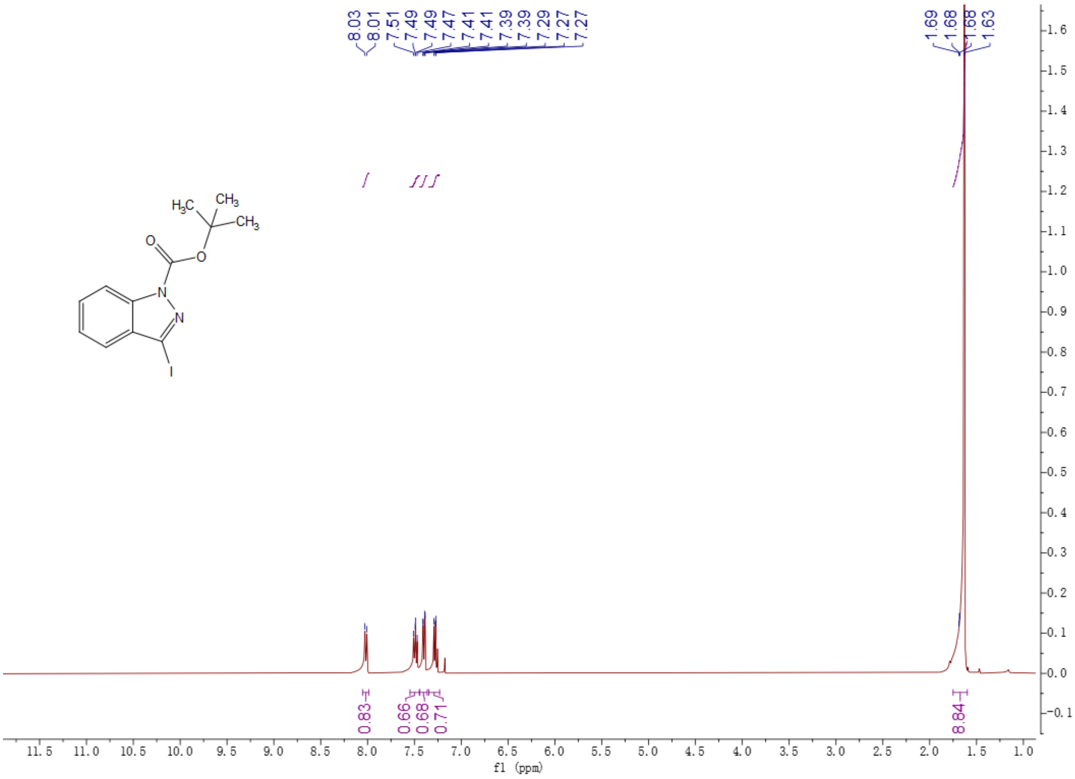

Figure S7. ^{1}H NMR spectrum of *tert*-butyl-3-iodo-1*H*-indazole-1-carboxylate (full scale).

S8. ^{1}H NMR spectrum of *tert*-butyl-3-iodo-1*H*-indazole-1-carboxylate (aromatic region)

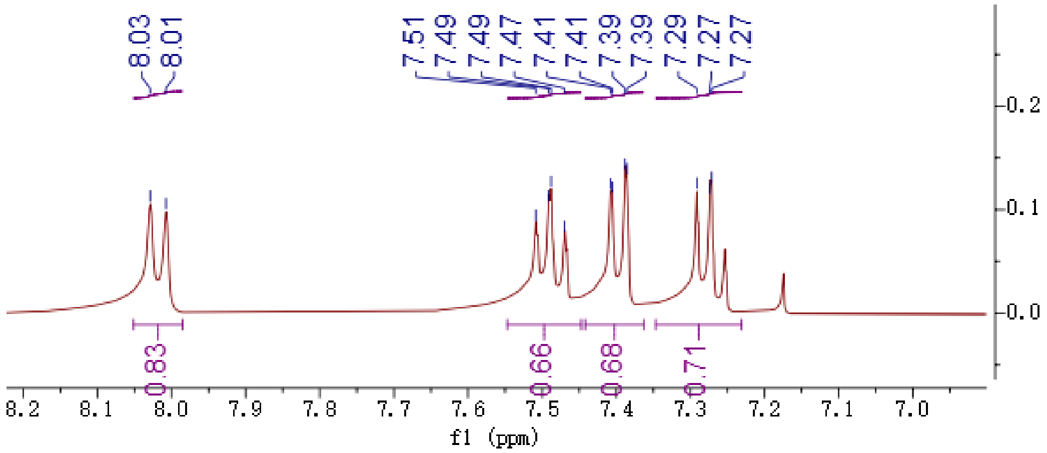

Figure S8. ^{1}H NMR spectrum of *tert*-butyl-3-iodo-1*H*-indazole-1-carboxylate (aromatic region).

S9. ^{1}H NMR spectrum of *tert*-butyl-3-(3-(methoxycarbonyl)phenyl)-1*H*-indazole-1-carboxylate (full scale)

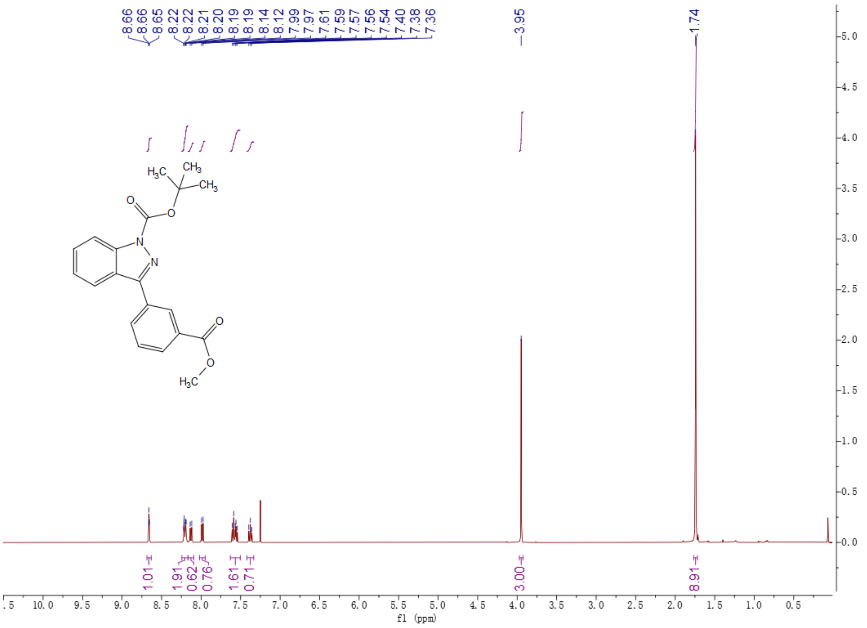

Figure S9. ^{1}H NMR spectrum of *tert*-butyl-3-(3-(methoxycarbonyl)phenyl)-1*H*-indazole-1-carboxylate (full scale).

S10. ¹H NMR spectrum of *tert*-butyl-3-(3-(methoxycarbonyl)phenyl)-1*H*-indazole-1-carboxylate (aromatic region)

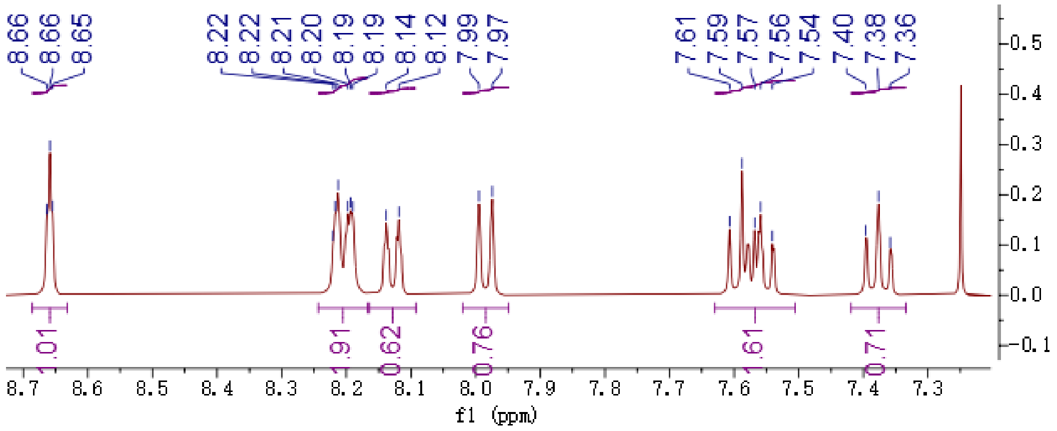

Figure S10. ¹H NMR spectrum of *tert*-butyl-3-(3-(methoxycarbonyl)phenyl)-1*H*-indazole-1-carboxylate (aromatic region).

S11. ^{1}H NMR spectrum of *tert*-butyl-3-(4-(methoxycarbonyl)phenyl)-1*H*-indazole-1-carboxylate (full scale)

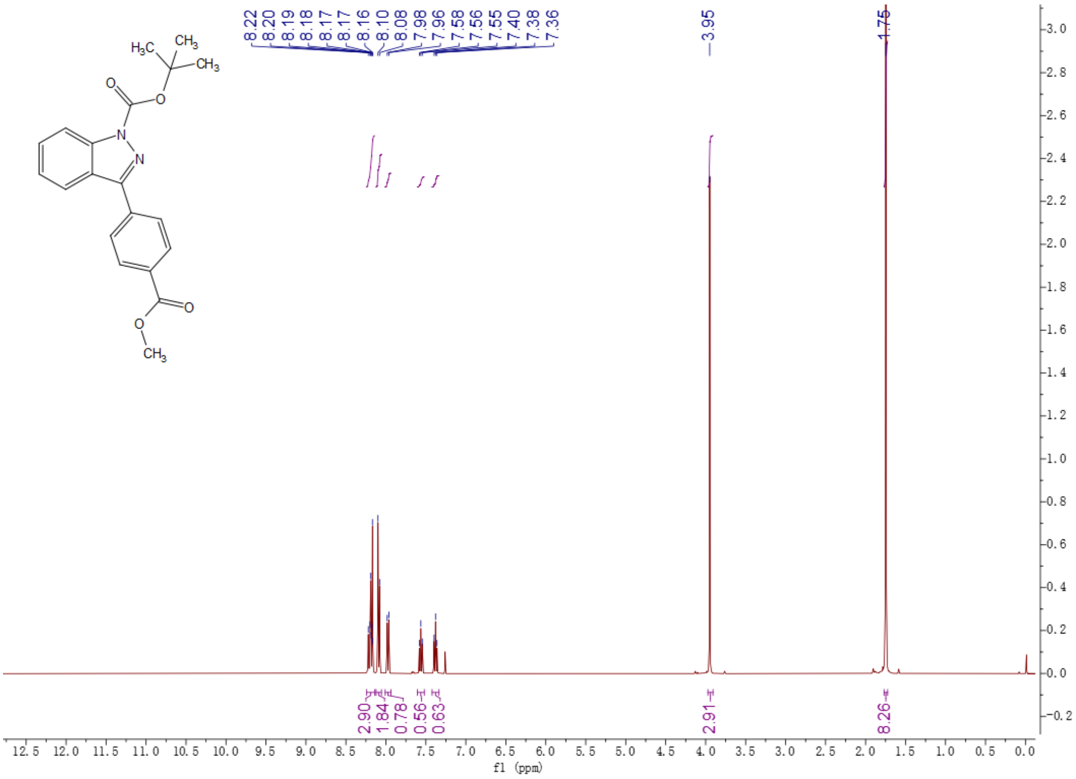

Figure S11. ^{1}H NMR spectrum of *tert*-butyl-3-(4-(methoxycarbonyl)phenyl)-1*H*-indazole-1- carboxylate (full scale).

S12. ^{1}H NMR spectrum of *tert*-butyl-3-(4-(methoxycarbonyl)phenyl)-1*H*-indazole-1-carboxylate (aromatic region)

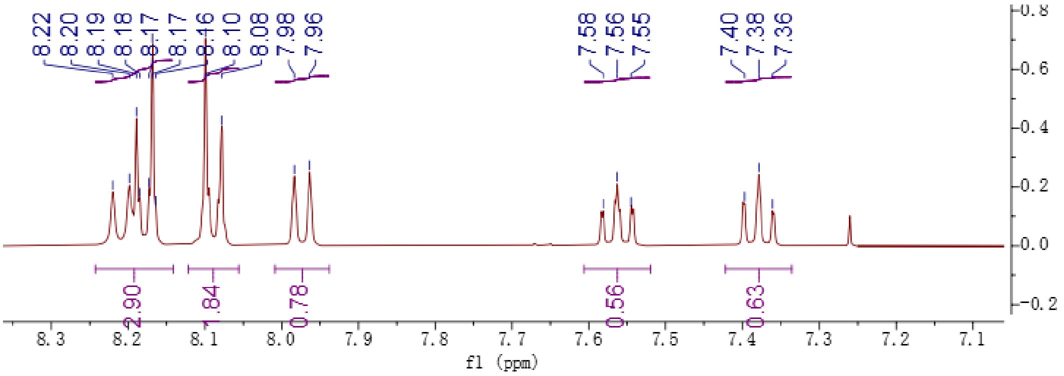

Figure S12. ^{1}H NMR spectrum of *tert*-butyl-3-(4-(methoxycarbonyl)phenyl)-1*H*-indazole-1- carboxylate (aromatic region).
